# EEG Features in Adolescent Patients with Borderline and Narcissistic Personality Disorder

**DOI:** 10.1192/j.eurpsy.2022.946

**Published:** 2022-09-01

**Authors:** A. Iznak, E. Iznak, E. Damyanovich, E. Krylova, A. Kuleshov, V. Kaleda

**Affiliations:** 1 Mental Health Research Centre, Laboratory Of Neurophysiology, Moscow, Russian Federation; 2 Mental Health Research Centre, Department Of Youth Psychatry, Moscow, Russian Federation

**Keywords:** adolescence, narcissistic personality disorder, borderline personality disorder, quantitative electroencephalography

## Abstract

**Introduction:**

Personality disorders (PD) in adolescence are widespread. It creates problems of social adaptation of patients and represents significant risk factors for auto-aggressive behavior, including suicidal one. The neurobiological basis and EEG markers of PD in adolescence have not been adequately studied.

**Objectives:**

The aim of the study was to reveal the EEG features and their correlations with clinical parameters in male adolescents with borderline personality disorder (BPD) and narcissistic personality disorder (NPD), possibly mediating some aspects of their clinical traits.

**Methods:**

28 BPD patients (301.83, by DSM-5) and 24 NPD patients (301.81, by DSM-5), as well as 24 healthy controls (HC) aged 16-25 years were enrolled in the study. HDRS-21 and HAM-A scales were used for quantitative assessment of patient’s conditions. Pre-treatment resting EEG was recorded, and EEG spectral analysis was carried out in 8 narrow frequency sub-bands. Descriptive statistics and correlation analysis of EEG and clinical data were performed.

**Results:**

EEG spectral parameters in BPD group did not differ significantly from those of HC. NPD group shows the EEG signs of more activated brain cortex than in both BPD and norm groups caused by decreased functional state of the anterior cortical regions. The structure of correlations between EEG parameters and clinical scores also differed between BPD and NPD groups.

**Conclusions:**

The data obtained suggests that these features of the brain activity may contribute to the disturbance of emotion regulation and of behavior control in adolescent patients with BPD and NPD, more pronounced in NPD group.

**Disclosure:**

No significant relationships.

